# Notch Signaling in Acute Inflammation and Sepsis

**DOI:** 10.3390/ijms24043458

**Published:** 2023-02-09

**Authors:** Nadia Gallenstein, Lucas Tichy, Markus Alexander Weigand, Judith Schenz

**Affiliations:** Department of Anesthesiology, Heidelberg University Hospital, 69120 Heidelberg, Germany

**Keywords:** Jagged, DLL, SIRS, infection, immune cells, immune response, Notch, therapy, inflammation, sepsis

## Abstract

Notch signaling, a highly conserved pathway in mammals, is crucial for differentiation and homeostasis of immune cells. Besides, this pathway is also directly involved in the transmission of immune signals. Notch signaling per se does not have a clear pro- or anti-inflammatory effect, but rather its impact is highly dependent on the immune cell type and the cellular environment, modulating several inflammatory conditions including sepsis, and therefore significantly impacts the course of disease. In this review, we will discuss the contribution of Notch signaling on the clinical picture of systemic inflammatory diseases, especially sepsis. Specifically, we will review its role during immune cell development and its contribution to the modulation of organ-specific immune responses. Finally, we will evaluate to what extent manipulation of the Notch signaling pathway could be a future therapeutic strategy.

## 1. Introduction

The Notch signaling pathway is a highly conserved regulative pathway that is present in all mammal cells and has its evolutionary origin in Drosophila melanogaster [1]. Notch signaling is crucial for a variety of processes from embryonic development to postnatal tissue homeostasis [2,3].

The plethora of functions regulated by Notch signaling becomes apparent when its role in the mammalian immune system is examined. In this context Notch signaling is important for differentiation as well as homeostasis of immune cells and is directly involved in the transmission of immune signals. A considerable body of evidence indicates that besides its well described role during cell differentiation, active Notch signaling also unfolds miscellaneous effects during various inflammatory events [4]. 

Notch Ligands like Delta-like 1 (DLL1) have the potential to activate cellular immune responses via the Notch pathway, resulting in the release of proinflammatory cytokines [5]. DLL1 is further involved in sepsis-induced endothelial damage leading to a loss of its barrier function [6]. Since the endothelium plays an important role for the recognition of pathogens but also for vascular integrity, DLL1 and other Notch ligands might contribute to the pathological host response during sepsis [7]. The Notch cascade is of rational interest for sepsis research because of its modifying capacity in T cell dysfunction and a possible association with sepsis-induced immunosuppression [8,9,10]. In line with that, a pathophysiological impact of Notch signaling on human monocytes is of noteworthy interest because it plays a pivotal role in the development of sepsis [11].

### 1.1. Notch Signaling Pathway

**Figure 1:**
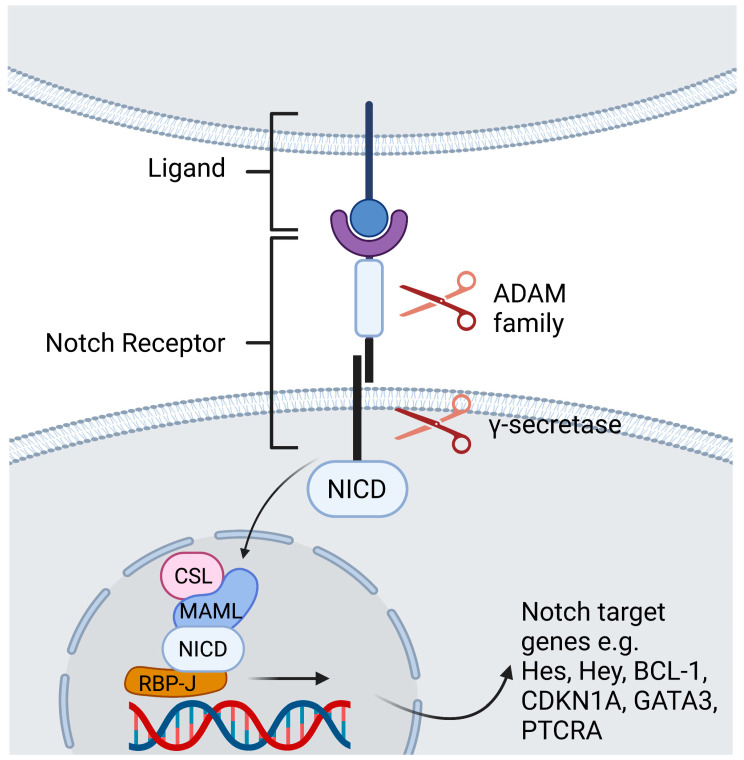
Contact dependent Notch signaling between cells. Ligand binding leads to a conformational change of the receptor. The first cleavage is arbitrated by the ADAM -family which leads to shedding of the extracellular domain. The second cleavage takes place inside the transmembrane domain and is catalyzed by a γ-secretase complex, that discharges the NICD to translocate to the nucleus. Within the nucleus, the NICD binds the DNA-binding protein RBP-J. Binding of NICD leads to transcription of the Notch target genes. Created with BioRender.com.

In mammals there are four Notch receptors (Notch1-4) and five Notch ligands [Jagged-1, Jagged-2, Delta-like 1 (DLL1), DLL3, and DLL4]. The ligands have both a membrane-bound and soluble form [2]. Both the receptor as well as its ligands are transmembrane proteins with abundant extracellular domains [12]. Ligand binding leads to a conformational change of the receptor and thus facilitates two consecutive proteolytic processes. The first cleavage is arbitrated by the A dys integrin and metalloproteases (ADAM)-family which leads to shedding of the extracellular domain. The second cleavage takes place inside the transmembrane domain and is catalyzed by a γ-secretase complex. The Notch intracellular domain (NICD) is cleaved and subsequently translocates to the nucleus to generate a transactivation complex [13]. This complex is composed of the deoxyribonucleic acid (DNA)-binding C-promoter-binding factor CBF-1 (CSL)/recombination signal-binding protein Jκ (RBP-J), a recombination signal sequence binding protein for Jκ genes) in mammals [14], and Mastermind-like protein (MAML) as a coactivator protein [15]. This process removes co-repressing complexes, mobilizes co-activators such as mastermind proteins, and ultimately leads to transcription of Notch target genes (Figure 1) [12]. The primary Notch target genes include two families of transcriptional factors hairy and enhancer of split (Hes), including *HES1* and *HES5*, and hairy/enhancer-of-split related with YRPW motif (Hey), including *HEY1* and *HEY2*. Other Notch target genes are B Cell lymphoma 1 Protein (*BCL-1*), cyclin dependent kinase inhibitor (*CDKN1A*), GATA binding protein 3 (*GATA3*), and Pre-T cell antigen receptor alpha (*PTCRA*) [5]. The interaction of Notch receptors with its ligands can be translationally modulated in the Golgi complex by O-linked glycosylation of the receptors. These post-translational modifications are initiated by the enzyme GDP-fucose Protein O-fucosyl transferase 1 (POFUT1), which adds fucose to small cystine-rich motifs called epidermal growth factor (EGF)-like repeats of the Notch extracellular domain. Additional sugar residues can be added to the fucose by glycosyltransferases, including members of the Fringe family proteins. In mammals, there are three Fringe enzymes referred to as Lunatic (Lfng), Manic (Mfng), and Radical Fringe [16]. These Fringe proteins provide for addition of N-acetylglucosamine residues to the glycan chain. Notch receptor glycosylation by Lfng and Mfng results in increased activation by DLL and decreased activation by Jagged ligands, while glycosylation by Radical Fringe enhances activation by all Notch ligands [17]. Besides this canonical Notch pathway, there are RBP-J independent non-canonical Notch signaling pathways [18]. These are reviewed extensively elsewhere [18,19,20,21]. 

### 1.2. Pathogenesis of Sepsis

A systemic inflammatory response syndrome (SIRS) is an over-excessive response of the body to a harmful stressor (surgery, acute inflammation, ischemia and reperfusion, malignancy, infection, or trauma). Acute-phase reactants are released and directly mediate autonomic, endocrine, hematologic, and immunologic changes. The immune cascade, which is activated to fight the infection, leads to a systemic, uncontrolled overactivation of pro- and anti-inflammatory processes. These massive inflammatory processes lead to organ dysfunction and, in the worst case, death. SIRS resulting from an infection (bacterial, viral, or fungal) is termed sepsis [22]. Sepsis is defined as a life-threatening organ dysfunction caused by a dysregulated host response to infection [23]. The syndrome involves physiological, pathological, and biochemical abnormalities [24]. On cellular and molecular levels, the pathogenesis of sepsis ultimately leads to (multi-)organ dysfunction, which is extremely complex. Among others, imbalance in inflammatory response, immune dysfunction, mitochondrial damage, coagulopathy, neuroendocrine immune network abnormalities, endoplasmic reticulum stress, and autophagy are involved [25]. Pathogen- or Damage associated molecular patterns (PAMPs and DAMPs), e.g., Lipopolysaccharides (LPS), activate immune cells through pattern recognition receptors that trigger transcription of type I interferons (IFNs) and proinflammatory cytokines. In a proinflammatory environment, macrophages differentiate into M1 or classic macrophages. In an anti-inflammatory environment, M2 or alternative phenotype macrophages arise predominantly [26]. 

In the past decades, the role of Notch signaling in macrophages during inflammation and infection has been increasingly well characterized [27,28,29]. Notch signaling has been shown to promote a pro-inflammatory microenvironment and control the macrophages’ pro-inflammatory responses in different inflammatory settings [30,31]. Other cell types, such as lymphocytes [32], endothelial cells [33], and smooth muscle cells [34] are known to express Notch receptors and thus also can influence the severity and course of the disease of sepsis. 

## 2. Notch Signaling and Lineage Cell-Fate Decision in Immune Cells

Human Notch receptor and ligands are expressed in CD34^+^ bone marrow (BM) hematopoietic stem cells (HSCs) and hematopoietic stem progenitor cells (HSPCs) [35]. In the lymphoid lineage Notch signaling is essential for T cell differentiation from HSPCs at different stages, both in the BM and thymus [36,37]. The target genes whose transcription is activated by Notch signaling at different stages of T cell development depend on the timing and microenvironment. Epigenetic remodeling which takes place constantly in thymocytes is related to it [38,39,40]. Notch signaling is thought to be critically involved in the binary cell fate decision of lymphocytes, in which either T or B cells develop at the expense of the other. As a pleiotropic process, Notch signaling has a variety of genetic targets depending on receptor-ligand combination, cell type, localization, epigenetics, and other stressors [41]. 

Several studies have investigated the role of Notch signaling in the recognition and modulation of innate and adaptive immunity [42]. There is much information available on the importance of Notch signaling in the host immune response and regarding the effects of Notch on direct or indirect modulation of the immune response. The interconnection between the Notch signaling pathway and the immune system is evident as there is a broad expression of Notch receptors and ligands in immune cells [2]. It is apparent that Notch ligands expressed on stromal cells in secondary lymphoid organs play a pivotal role in immune response regulation. Fibroblasts that express the ligands DLL1 and DLL4, on the other hand, drive different processes of immune cell differentiation [43].

### 2.1. Notch in Hematopoietic Stem Cell Development and Homeostasis

Over the last 20 years [35,36,37] it was established that Notch signaling is directly linked to hematopoietic cell formation independently of its role in arterial development [44,45,46,47]. Thus, it is essential for definitive hematopoiesis in the developing embryo. Whether Notch signaling plays a similar role during the generation or maintenance of HSC in the adult BM compartment is still convoluted [48]. Jagged-1-induced Notch signaling was shown to regulate HSC homeostasis [49]. On the contrary, however, there is evidence that canonical Notch signaling is dispensable for HSC homeostasis in the BM. In adult HSCs, the intensity of Notch signaling appears to be too low to translate into a detectable physiological function. Thus, the avoidance of high levels of Notch signaling in hematopoietic progenitors is thought to be a carefully regulated phenomenon that has an important physiological role in preventing ectopic development of T cells and suppression of B lineage development in the BM [50].

### 2.2. Lymphoid Cells 

#### 2.2.1. T Cells 

The role of Notch signaling during thymic T cell lineage commitment and maturation is the best-studied aspect of Notch signaling’s impact on hematopoiesis. BM progenitors constantly reach the thymus via the bloodstream. Canonical Notch1 signaling leads the bipotent early thymic progenitor to develop into a T cell before emigrating to the periphery [2]. Notch1 is a key receptor expressed on thymus-seeding cells responsible for T cell lineage commitment. Inactivation or disruption of Notch1 results in impaired T cell development [51,52,53,54,55,56,57]. It has recently been shown that *HES1* and *HES4* are Notch1-dependently induced during early human T-cell development. Importantly, knockdown of HES1 or HES4 significantly reduces human T-cell development [58]. 

Based on their capacity to support complete development of mature T cells from BM precursors in vitro, DLL1 and DLL4 have been endorsed as potential Notch1 ligands for T cell fate specification [59,60,61]. The interaction of DLL4-expressing thymic epithelial cells and thymus-seeding Notch1-expressing hematopoietic progenitors is essential for T lineage commitment. Maturation of thymocytes to the CD4^+^CD8^+^ stage induces downregulation of DLL4 on cortical thymic epithelial cells [62]. However, it is not yet understood to what extent this is fundamental for positive or negative selection. 

Pre-T cell receptor (TCR) signaling is essential for β-selection and further thymocyte development. While thymocytes undergo β-selection, Notch assures survival by regulating glucose metabolism [63,64]. In vitro experiments suggest that successful CD4^−^CD8^−^ into CD4^+^CD8^+^ transition proceeds symbiotic signaling of both Notch and pre-TCR [65]. Notch1 as well as Notch3 can directly activate the transcription of the pTα gene. Therefore, a direct crosstalk between Notch signaling and the pre-TCR is assumed [66,67]. Compared to Notch1, Notch3 expression levels are significantly higher in CD4^−^CD8^−^ and CD4^+^CD8^+^ cells [68,69]. Notch3 expression is preferentially upregulated in CD4^−^CD8^−^immature cells prior to their transition to CD4^+^CD8^+^ cells and subsequently downregulated during transition [68]. As mentioned earlier, the transition is controlled by the pre-TCR signaling pathway and is characterized by activated NF-κB [70,71], proposing possible interactions between Notch3-, pre-TCR-, and NF-κB-induced pathways. Consistent with that, Lck promoter-driven Notch3-IC (Lck-Notch3-IC) transgenic mice display a dysregulated early T cell development, by the significant expansion of CD25^+^ involving the impairment of the pre-TCR selection [72].

Earlier observations suggest that Notch1 may play a more general role in promoting the maturation of CD4^+^CD8^+^into both the CD4^+^ and CD8^+^ single lineages. *Deltex*, *Meltrin β*, *Ifi-204*, and *HES1* are transcriptionally regulated by Notch1 signaling in thymocytes. These genes are expressed at low levels in CD4^+^CD8^+^ and high levels in CD4^+^ and CD8^+^, suggesting that Notch signaling is upregulated during the double positive to single positive transition [73]. In later stages of T cell development, *GATA3*, master regulator for both T cell development and for Th1/2 lineage decision, is a direct Notch target gene [74,75].

In peripheral T cells, Notch receptor expression is linked to T cell activation, proliferation, and cytokine production. TCR activation in vitro leads to upregulated Notch1 expression [76]. Notch signaling is also involved in the differentiation of naïve CD8^+^ T cells to cytotoxic T lymphocytes [77]. 

In summary, Notch1- respectively Notch3- mediated signaling is crucial for the development of a functional pre-TCR and as soon as thymocytes pass the β-selection, the pre-TCR assures the transcriptional repression of Notch1, a mechanism that is apparently important to avoid the oncogenic properties of Notch signaling [78]. 

#### 2.2.2. B Cells

In addition to T lymphocyte differentiation regulation, Notch signaling is participating in the maturation of B lymphocytes, particularly in the specification of the two major subsets, follicular and marginal zone B cells (MZB) in the spleen. Follicular B cells are the most abundant subset and are circulating cells involved in the T cell-dependent immune response. MZB cells are localized in the outer region of the splenic white pulp. By eliciting T cell-independent antibody responses, they provide an important defense mechanism against pathogens [55]. B lineage progenitors from BM develop into both MZB cells and follicular B cells in the spleen. Immature B cells rearrange heavy- and light-chain immunoglobulin genes to express a B cell receptor (BCR) at the cell surface. Further, B cell maturation continues through brief transitional stages, ultimately leading to the differentiation in the spleen. Several factors like tonic BCR signaling and B cell-activating factor (BAFF), canonical nuclear factor-κB (NF-κB) signaling, or regulatory enzymes determine the fate of immature B cells in the spleen as follicular B cells or MZB [79]. Development of MZB, but not follicular B cells, requires Notch signaling [80]. The interaction between Notch2 and DLL1 is critical. The signaling strength from this interaction thereby controls the development rate of MZB [51,81,82,83]. Although DLL1 is the relevant ligand, it remains elusive, which of the DLL1-expressing cells are the most necessary ones. Various non-hematopoietic cells [59] including endothelial cells located in the red pulp and marginal zone of the spleen [84], express DLL1. It can be assumed that endocytosis of DLL1 by these endothelial cells is required for efficient signal transduction via Notch2 to MZB or their progenitors [85]. Another modulator of Notch signaling during MZB development is the Fringe family of glycosyltransferases. Fringe raises Notch:DLL ligand interaction. The two family members Lunatic fringe and Manic fringe act synergistically to enhance the rather weak interaction between Notch2 on MZB cells or their precursors and DLL1 expressed on endothelial cells [84]. Scheikl et al. linked the putative adaptor protein SLy1 and the activity of the Notch pathway in MZB. In Sly1-/- mice, the expression of *RBP-J*, *HES1,* and *HES5* was markedly reduced in MZB but not in follicular B cells. The reduced expression of *RBP-J* is associated with an impaired Notch activity in *Sly1*d/d MZ B cells [86]. Furthermore, there is evidence that Notch signaling favors the generation of MZB by down-regulating E protein activity. E proteins are well known to play crucial roles in immunoglobulin gene expression and receptor editing. Activation of Notch signaling promotes the degradation of E2A proteins triggered by their ubiquitination. Gene expression of the inhibitory molecule Id2, a molecule inhibiting E protein function, and the ankyrin-repeat SOCS box-containing protein 2 ((Asb2), capable of facilitating E2A ubiquitination) in MZB is increased by Notch signaling. Excessive amounts of Notch1 stimulate the MZB differentiation. More interestingly, by gaining E protein function the effects of Notch1 are reversed. Taken together, it is evident that Notch regulates peripheral B cell differentiation, at least in part, through opposing E protein function [87].

Antibody-secreting cells (ASC) can develop from B cells in either T cell dependent or T cell independent immune responses. T cell-independent responses tend to generate short-lived ASC that remain proliferative plasma blasts. T cell-dependent germinal center reactions produce longer-lived, non-proliferative, and antibody-secreting cells (fully differentiated plasma cells). Long-lived plasma cells contribute in a significant way to immunological memory since they continue to secrete high affinity isotype-switched antibodies over decades [88]. The involvement of the Notch pathway has been described for ASC differentiation. The effects of co-culturing B cells and the Notch ligand DLL1 have been studied in detail. B cell activated T-cells, with LPS in the presence of DLL1, develop a higher number of ASCs, producing higher antibody titers without affecting B cell proliferation [89]. Deletion of Notch1 reduces B cell antibody secretion in response to LPS stimulation [90]. DLL1 increases isotype switching and changes the pattern of secreted antibody isotypes in stimulated B cell cultures [91]. The effects of DLL1 on antibody secretion depend on Mastermind Like Transcriptional Coactivator 1 (Maml1), a Notch co-activator. Many studies suggest that follicular B cells upregulate Notch1 during activation and that thereupon, Notch1 expression promotes ASC generation [92,93]. 

To summarize, Notch is essential for the development of definitive hematopoiesis during embryogenesis. On the other hand, it is dispensable for the maintenance or homeostasis of adult HSCs under physiological conditions. Furthermore, it is necessary for T cell lineage commitment and early stages of thymocyte as well as for MZB development. All these developmental aspects of Notch function are mediated by Notch receptor ligand pairs and canonical signaling. 

### 2.3. Myeloid Cells

The role of Notch signaling in myeloid cell differentiation is not yet conclusively elucidated. Most in vitro experiments have shown that active Notch1 initiated signaling expands the stem cell compartment but blocks or delays terminal myeloid cell differentiation [94,95,96]. However, there are several reports of an opposite role of Notch signaling in myeloid cell differentiation. Caton et al. shifted the differentiation of a murine pre-B cell line towards a myeloid phenotype by activating Notch signaling through ligation of Jagged-1 [97]. Human CD34^+^ progenitor cells can also be differentiated into myeloid cells in this way [98]. Loss-of-function experiments, on the other hand, have shown that differentiation of myeloid cell lineages proceeds normally [52,99]. However, the exact nature of Notch effects remains controversial. Existing findings can be split into two groups: one demonstrating a critical role of Notch in sustenance of progenitor cells and blocking of terminal differentiation of myeloid cells, and the other showing requirements of Notch signaling for differentiation of mature myeloid cells. It appears that impact of Notch signaling on myeloid cell differentiation depends on the stage of myeloid cell differentiation when Notch activation is triggered, the presence of specific cytokines, and on whether activation of Notch signaling was triggered by soluble or immobilized ligands. 

#### 2.3.1. Dendritic Cells

Dendritic Cells (DC) differentiate from myeloid progenitors in the BM and are derived from an HSC that can give rise to two distinct lineages [100]. They both express the DC marker CD11c and can be further distinguished into primarily resident cells in lymphoid tissues and cells that localize preferentially in the thymus [101]. Murine splenic CD11c^+^ DC express transcripts for Jagged-1 and Jagged-2 but are low in DLL1. Conversely, thymic CD11c^+^ DC expresses high levels of Jagged-2 but lower levels of Jagged-1 and DLL1 [102,103]. Although the data suggest that Notch ligand expression is widespread amongst DC and macrophage populations, virtually nothing is known about the physiological role for the individual ligands in the function of these mature cell lineages [102].

In vitro experiments using primary cell culture with human peripheral blood monocytes suggest that Notch signaling helps regulate the macrophage/DC cell fate choice. DLL1-induced Notch signaling could greatly increase the proportion of monocytes that differentiate into DC when cultured in the presence of either granulocyte-macrophage colony-stimulating factor (GM-CSF) and tumor necrosis factor TNF [104]. The type of cytokine present during the time in which a precursor cell receives a Notch signal could also have important implications on the differentiation process. This is illustrated by the finding that DLL1-induced Notch signaling together with macrophage colony-stimulating factor (M-CSF) can induce apoptosis of monocytes, whereas the delivery of a Delta:Notch signal together with GM-CSF protects cells from death. Therefore, the combinatorial effects of Notch and cytokine-induced signaling on immature cells can have distinct influences on the outcome of hematopoietic cell differentiation. Cell fate decisions, such as those that regulate macrophage versus DC lineage choice, will normally be made within defined microenvironmental niches and will be influenced by the presence of cytokines and Notch ligands present on stromal cells [105]. Most of the experiments demonstrate the induction of differentiation of plasmacytoid DC (pDC) when Notch signaling is activated by DLL1. In contrast, “loss of function”-experiments result in Notch signaling that either has no effect or inhibits pDC development [106].

#### 2.3.2. Monocytes

Monocytes can be differentiated into M1- type macrophages with GM-CSF, Interferon (IFN)-γ, lipopolysaccharide (LPS) and other microbial products or into M2-type macrophages with M-CSF, interleukin (IL)-4, IL-13, IL-10, and immune-suppressive agents (corticosteroids, vitamin D3, prostaglandins), respectively [107,108]. Peripheral blood monocytes express relatively high amounts of Notch-1 and Notch-2 [105]. To evaluate the potential role of Notch signaling in monocyte subset regulation, Gamrekelashvili et al. sorted Ly6C^high^- and Ly6C^low^ monocytes from the BM and determined their Notch-related gene-expression patterns. Compared with Ly6C^high^ monocytes, Ly6C^low^ monocytes have lower expression of *Notch1*, but is comparable *Notch2* expression. Furthermore, *Hey2* and *Hes1*, are markedly induced in Ly6C^low^ monocytes. Analysis of the human non-classical CD16^+^ monocytes, which are considered equivalents of mouse Ly6C^low^ monocytes, also express higher levels of *HES1* compared with the classical CD14^+^ monocytes [109]. It is indicated that Notch signaling affects the differentiation of macrophage precursors [104]. Fung et al. demonstrated that DLL4 increased in macrophages exposed to proinflammatory stimuli such as LPS, IL1β in a Toll-like-receptor (TLR) 4 -and NF-κB-dependent manner. Coculture of macrophages with DLL4 expressing cells triggered Notch proteolysis and activation, increased the transcription of proinflammatory genes, and resulted in activation of Mitogen-activated protein kinases (MAPK), Protein kinase B (Akt), and NF-κB pathways. Combined with the results of DLL4 presence within macrophages in atherosclerotic plaques, these in vitro data clarify the Notch signaling implications for inflammation by macrophages [30]. The role of Hes1 on gene regulation in primary macrophages and in inflammatory conditions in vivo is inhibiting inflammation and especially neutrophil-mediated responses by controlling production of macrophage-derived chemokines, e.g., Hes1 suppresses the production of CXCL1. The inhibitory effects of Hes1 are highly restricted to a small subset of genes in the macrophage inflammatory transcriptome [110]. 

Xu et al. suggested that RBP-J enhances TLR4-induced expression of key mediators of M1 macrophages and thus of innate immune responses. Notch-RBP-J signaling controls the expression of the transcription factor IRF8 that induces downstream M1 macrophage-associated genes [111]. Notch and TLR pathways cooperatively activate Notch target genes and increase the production of TLR-induced cytokines in murine macrophages [30,112,113]. Furin, a calcium-dependent serine protease, has a dual role in inflammation-driven Notch regulation. On the one hand, it cleaves Notch receptors itself, and on the other hand, activates other proteases involved in Notch signaling, including ADAM and ADAM1. This leads to Notch-dependent TLR activation [114]. It is also indicated that Notch signaling plays an important role in inflammatory disorders [115,116]. In murine macrophages, LPS-induced Jagged-1 is expressed in a C-Jun-N-terminal Kinase (JNK)-dependent manner. Notch target genes were upregulated by early Notch-independent activation followed by delayed Notch-dependent activation after LPS stimulation [117]. Conversely, M2-like tumor-associated macrophages (TAMs) exert lower levels of Notch pathway activation in mouse tumor models. Forced activation of RBP-J-mediated Notch-signaling in macrophages augmented their antitumor capacity which regulated M1 versus M2 polarization [118]. 

### 2.4. Endothelial Cells

Endothelial cells (ECs) are a heterogeneous cell population that participates in many physiological processes. They are dynamic cells that respond to changes in the extracellular environment. ECs actively take part in both innate and adaptive immune responses. ECs are one of the first cells detecting foreign pathogens and endogenous metabolite-related danger signals in the bloodstream [119]. Regulation of cell fate decisions is a hallmark of Notch signaling, and in the vasculature, Notch promotes stalk and tip cell specification. A broad range of data indicates that Notch is required for vascular stabilization and differentiation of the vascular tree through suppression of endothelial cell proliferation and stabilization of cell–cell junctions [120,121,122]. During the development of the vascular sprout, it is characterized by a leading ‘tip’ cell and ‘stalk’ cells. Tip cells express high levels of DLL4 that can activate Notch1 in the stalk cells to enforce differential gene expression. Bone morphogenetic proteins (BMP), part of the TGFβ superfamily, bind receptors to induce nuclear translocation of Suppressor of Mothers against Decapentaplegic (SMAD) transcription factors and regulate vessel growth. Endothelial cell responsiveness to these BMP ligands is regulated by Notch. Notch determines responsiveness by regulating the cell-intrinsic BMP inhibitor SMAD6, which affects BMP responses upstream of target gene expression [123]. Both the endothelial and non-endothelial derived vascular endothelial growth factor (VEGF) lead to an increase of DLL4 in tip cells to activate VEGF receptor (VEGFR2) [124,125]. In stalk cells, NICD translocates to the nucleus and regulates gene expression [126]. A consequence of Notch-initiated Notch expression is suppressed proliferation and dosing of the signaling mediated by bone morphogenetic proteins (BMP). Regulation of new sprouting during vascular expansion depends on cooperation of BMP signaling, Notch signaling and, VEGF signaling. Differential expression patterns of those pathways are required to enable sprouting of new vessels. In contrast, synchronized variations of the pathways favor vessel enlargement and disrepute branching. In adult vessels, Notch is responsible for maintaining endothelial quiescence and junctional integrity [127]. 

The association of Notch expression with arteries was the first finding ever that linked this signaling pathway to blood vessels [128]. Inactivation of Notch1 in zebrafish impaired arterial differentiation [129], underlining the essential need for Notch1 in arterial specification. More recently, transgenic lines visualized the constant need for Notch signaling for the maintenance of arterial fate [130]. Although this observation was made in the vascular development in zebrafish, recent publications have highlighted the requirement of continuous Notch signaling for arterial specification in mammals [122,131,132]. In the regulation of endothelial cell fate, biomechanical forces are of great relevance [133]. Notch1 turned up as a mechano sensor responsible for both promoting and maintaining arterial homeostasis [121,122]. Arterial Notch1 expression is continued by high shear stress leading to suppression of cell cycle and retention of arterial identity. Absence of Notch encourages arteriovenous shunts and convoluted vascular networks [134]. 

In lymphatics, Notch exerts slightly different effects. Notch1 maintains lymphatic specification and limits lymphatic endothelial cell differentiation from veins [135]. During lymphatic vessel sprouting, Notch1:Dll4 signaling is required for postnatal lymph angiogenesis. Inhibition of Notch signaling with function-blocking antibodies decreases lymphatic density [136]. In contrast to arterial endothelium, fluid flow forces in lymphatic vessels reduce Notch activity and enhance lymphatic endothelial sprouting [137]. Reduction in Notch signaling activates both blood and lymphatic endothelial sprouting and shows that Notch activity is modulated by shear stress.

## 3. Notch Signaling in Inflammatory Diseases

Considering that Notch signaling, as discussed in the previous section, regulates a multitude of processes in the human immune system, in particular the differentiation of progenitor cells into mature effector cells, its role in the pathophysiology of inflammatory disorders comes as little surprise. The spectrum of diseases is as broad as the cellular functions controlled by Notch signaling. In various types of cancer [138], cerebrovascular diseases [139], and inherited disease syndromes [140], Notch signaling has been found to exert a detrimental impact as well as in inflammatory diseases such as rheumatoid arthritis [141], systemic lupus erythematosus (SLE) [142,143], systemic sclerosis (SSc) [144], primary biliary cirrhosis [145], and atherosclerosis [146]. In addition, it also contributes decisively to the coordination of the immune response to viral and bacterial infections [29,147]. Due to the broad range of these diseases, only a selection will be discussed in this section. The focus will be set on active Notch signaling during inflammatory events.

### 3.1. Notch Signaling in Leukemia and Cancer

Notch signaling as an important part of immune regulation was first described in a disease context. Aster et al. discovered that the *Notch1* gene leads to T-lineage acute lymphoblastic leukemia (T-ALL) due to (7;9) chromosomal translocation of *Notch1* to the *TCR* loci, inducing the expression of truncated forms of *Notch1* [148,149]. The truncation of the Notch1 extracellular domain enables constitutive production of NICD1 in the absence of ligand binding [150,151]. Additionally, activating mutations of Notch3 have been identified by screening primary T-ALL tumors and orthotopic patient-derived xenograft models, even in the absence of activated Notch1 [152].

Since then, Notch signaling has been found to be associated with both pro- and anti-tumorigenic functions in various types of cancers, depending on tissue and cell type [18]. The role of Notch as an oncogene is well characterized for many lymphoid malignancies such as T-ALL, B-chronic lymphocytic leukemia, and splenic marginal zone lymphoma. In contrast, there is increasing evidence that Notch signaling acts as a tumor suppressor in myeloid malignancies [41]. However, in solid tumors such as breast cancer, lung adenocarcinoma, hepatocellular cancer, ovarian cancer, and colorectal cancer activation of Notch has been identified to be oncogenic [153].

### 3.2. Notch Signaling in Autoimmune Diseases

Rheumatoid arthritis is an autoimmune disease that primarily affects joints and has a prevalence of approximately 1% of the worldwide population [154]. The impact of Notch on arthritogenic inflammation is multifaceted but governs inflammatory events like endothelial activation, pathologic angiogenesis, as well as leukocyte recruitment, activation, and function. Notch1-initiated signaling participates in hypoxia-induced angiogenesis and conceivably also in VEGF/angiopoietin 2 (VEGF/Ang2)-induced expression of IL-6, IL-8, and Matrix metalloproteinases (MMP) 2 and 9 [155]. Endothelial DLL1 modulates Notch2-mediated differentiation of monocytes involved in both initiation and progression of experimental arthritis [156]. 

SLE is a systemic autoimmune disease affecting different organ systems due to a deposition of immune complexes activating the complement system [157]. Cleaved Notch1, cleaved Notch2, and Jagged-1 are expressed on podocytes in protein uric nephropathies including lupus nephritis, one of the most serious manifestations of SLE [142]. Stronger mechanistic data revealed that Notch3 affects the progression of nephritis by promoting migration and pro-inflammatory pathways [158]. In line with these findings, constant Notch activation results in podocyte death and it is suggested that Notch acts as a regulator of regeneration in glomerular disorders [159]. 

SSc is a chronic fibrotic disease of unknown etiology that involves the skin, and diverse internal organs [144]. The resulting fibrosis disturbs the physiological structure of the affected tissues, disrupts proper organ function, and is the major cause of death in SSc patients [144,160]. The Notch pathway is thought to be implicated in the fibrosis that characterizes SSc. Indeed, in the lesioned skin of SSc patients and in their fibroblasts, activated Notch1 can be found [161,162]. Mice with reactive oxygen species (ROS)-induced SSc also display elevated levels of NICD, overexpression of the ligand Jagged-1, and increased transcription of the target gene Hes-1 in the skin and lungs [161]. The Notch pathway is activated in SSc and inhibition of Notch signaling with the γ-secretase inhibitor DAPT exerts potent anti-fibrotic effects in this preclinical model. 

### 3.3. Notch Signaling in Chronic Inflammation

There are many reports of Notch dysregulation in clinical samples from patients with different chronic inflammatory diseases. In colonic mucosal biopsies from patients with ulcerative colitis, transcription levels of *Notch1* and *Hes1* were significantly elevated [163]. A gene expression analysis revealed that Jagged-1 is expressed on endothelial cells from patients with giant cell arteritis, but not on endothelial cells from healthy individuals. Moreover, Notch1 was up regulated on circulating CD4^+^ T cells in these patients [164]. Likewise, in patients with asthma, circulating CD4^+^ T cells have been found to have higher *Notch1* and *Notch2* expression levels [165]. Higher levels of active Notch1 have also been observed in human appendix inflammation endothelial cells [166]. 

Atherosclerosis is an inflammatory disease characterized by the passive accumulation of lipids within artery walls [167]. Modified low-density lipoproteins (LDL), chronic infection, free radicals, or other factors cause a chronic inflammatory process involving the arterial endothelium [168]. Recently it has been shown that Notch signaling is activated in human aortal luminal endothelial cells at atherosclerotic lesions and modulates atherosclerosis by controlling macrophage polarization [169] into a proinflammatory phenotype [170]. Binesh et al. demonstrated in a rat model that enzymatic inhibition of NICD translocation by Diosgenin (a phyto steroid sapogenin) and γ-secretase inhibitor DAPT in differentiating macrophages leads to a significantly decreased NICD expression while at the same time the macrophage marker MAC387 is downregulated [171]. In vitro vascular inflammation models revealed that in different endothelial cells TNF promotes apoptosis through a downregulation of Notch activity. Additionally, it results in a phenotypic switch where Notch4 is replaced by Notch2. Further, Quillard et al. proved a relationship of Notch signaling, caspase activation, and apoptosis in a rat vascular inflammation model [172]. 

## 4. Notch Signaling in Systemic Inflammation and Sepsis

### 4.1. Molecular Mechanisms of Notch Activation by Inflammatory Stimuli 

A wide variety of proinflammatory stimuli including TLR ligands and cytokines are capable of activating Notch target gene expression in myeloid cells. NF-κB signaling is activated by both proinflammatory cytokines and TLR ligands and has been shown to interact with the Notch pathway in many systems [173]. Undoubtedly, TNF and TLR-induced Notch target gene expression is often dependent on inhibitor of NF-κB kinases (IKKs) [113,174], which are required for NF-κB activation by proinflammatory stimuli. MAPKs [113,175], a family of serine/threonine protein kinases, are key regulators of inflammation and have also been described as mediators of Notch pathway activation. Three complementary systems are known that explain NF-κB-mediated activation of canonical Notch target genes: The first is cooperation of transcription factors. The NICD has been observed to directly interact with NF-κB subunits and promotes transcription [176]. The second is the release of inhibitory molecules. In resting cells for example, inhibitor of NF-κB (IκB) is bound to the promoter regions of Hes1 [177]. The third is chromatin modifications. TNF and TLR ligand induced Hes1 gene transcription has been linked to an upregulation of positive histone marks and acetylation of histone H3 at the Hes1 promoter [113,174,177]. Both IKKs and MAPKs mediate inflammatory signaling-induced chromatin modifications at the Notch target gene loci [113,175,177]. Therefore, NF-κB and MAPK signaling seems to play a critical role in mediating Notch target gene activation by inflammatory stimuli. 

In viral infections the participation of Notch signaling can be essential for the development of an IFN-mediated response. This controls and limits viral replication. For hepatitis viruses, however, it has also been demonstrated that the Notch pathway can be regulated by the virus, which in turn can promote the disease [42].

In various bacterial infection studies, potential cross talks with Notch have been revealed. For example, Notch1 has been associated with the modulation of antimicrobial and inflammatory responses in *P. gingivalis* infections [178], and the modulation of suppressor of cytokine signaling 3 (SOCS3) and cyclooxygenase 2 (COX-2) expression [147,179]. Modulation of monocyte and CD4^+^ T cell function and activation [180,181], and Th17/Th2 response [182] were also observed. In *Helicobacter pylori* infections as well as in *Mycobacterium leprae* infections, modulation of the Th1 response has also been determined [183,184]. In a secondary analysis of a prospective cohort study of patients after liver transplantation, patients with bacterial infection had elevated DLL1 levels compared with patients without infection. Hence, the Notch ligand is useful for early detection of a broad spectrum of bacterial complications [185]. DLL1 is characterized by a high robustness in non-infectious inflammatory reactions and is therefore also suitable as a biomarker for the diagnosis of sepsis [11].

### 4.2. Notch Signaling in Sepsis

Dysregulated immune responses to infection in an immuno-compromised state together with vascular dysfunctions are the predominant cause of death in sepsis [186,187]. Circulatory failure in sepsis is characterized by headstrong hypotension and vascular hypo reactivity to clinically applied vasoconstrictors leading to multi-organ dysfunction. Although the pathophysiological understanding of sepsis has increased substantially in recent years, sepsis is still reported to be the leading cause of death in seriously ill patients, and the incidence of sepsis is increasing every year [188,189]. As discussed above, among other effects, Notch signaling alters TLR-driven inflammation and modulates monocyte and macrophage cell fate decisions in inflammation. Therefore, Notch signaling exerts a tremendous impact on the development or progression of sepsis [190]. 

To date, only a few studies have systematically explored closely this impact on sepsis progression. Recently Schneck et al. described soluble DLL1 (sDLL1) as a biomarker that discriminates sepsis from surgery-induced systemic inflammation within the first 24 h on intensive care unit (ICU). They could further assign a high specificity and sensitivity for acute kidney injury (AKI) detection to sDLL1 plasma levels. After cardiopulmonary bypass (CBP) however, sDLL1 levels exceeded the levels of abdominal surgical patients. Secondly, the authors report a strong positive correlation between sDLL1 and plasma creatinine and urea concentration as well as a negative correlation to the glomerular filtration rate (GFR), suggesting that CPB-induced AKI causes the increased plasma sDLL1 levels [191]. Whether the soluble forms of Notch ligands induce the same effect as the membrane-bound ones, however, has not been truly clarified. Studies published some time ago came to partly contradictory results: Using a recombinant, secreted form of DLL1, Hicks et al. demonstrated that pre clustering is required for Notch 1 to be internalized and downstream signaling to be activated. Interestingly, pre clustering with both a limited or excess amount of DLL-Fc-fusion protein does not result in activation of the intracellular signaling cascade. This suggests that ligand binding is necessary but not sufficient for activation of Notch signaling [192]. Alternatively, prior immobilization of the soluble ligand to activate Notch signaling and elicit the biological responses may be necessary as shown for myoblast differentiation, transactivation of human bone osteosarcoma epithelial cells, or during mouse HSC and progenitor cell proliferation and maturation [94,193]. However, other studies have concluded that the soluble forms can lead to activation, albeit weaker. In addition, the intracellular signaling cascade, particularly cleavage of the intracellular domain, appears to be altered compared to activation by membrane-bound forms [194]. Activation by soluble ligands can induce undesirable, pathological changes under some circumstances [124,195]. In the postnatal mouse retina, sDll4-Fc leads to several characteristic abnormalities in the developing retinal vasculature. Most notably, enhanced angiogenic sprouting and increased proliferation of endothelial cells was observed, resulting in the formation of a denser and more highly interconnected superficial capillary plexus [124]. Finally, antagonizing or blocking effects were also observed when soluble and full-length ligand forms were compared. Soluble forms of Notch ligands normally expressed on differentiating neuroblasts can inhibit neurogenesis in neural crest stem cells (NCSC). In isolated rat NCSC, sDLL1-Fc can inhibit neuronal differentiation. Contrary to expectation, withdrawal of sDLL1-Fc does not allow NCSC to resume neuronal differentiation. Rather, transient exposure to the soluble ligand results in a rapid and irreversible loss of neurogenic capacity accompanied by glial differentiation. [195]. In a mouse tumor model, a soluble form of DLL4 (D4ECD-Fc) blocked tumor growth by interfering with vascular function despite increased tumor vessel density [196]. In mouse fibroblasts, both sDLL1 and sJagged-1 act as Notch signaling antagonists [197]. The effect of a soluble ligand can vary depending on the tissue and form in which it binds, e.g., additionally clustered or immobilized. From weak activation to complete blocking of intracellular signal transduction to opposite effects compared to the membrane-bound forms, all effects are possible. Soluble ligands can also compete with membrane-bound ligands for Notch binding and thus modulate their effect. Precisely which of these effects are triggered by the increased levels of sDLL1 in sepsis needs to be the subject of future investigation.

Moll et al. cocultured HUVEC and blood. Here, sDLL1 led to endothelial cell activation and a loss of the endothelial barrier function by destruction of the structure. Blocking of DLL1-receptor binding and Notch signaling during LPS challenge partly prevented this endothelial barrier loss [6].

More recently, Liu et al. showed that LPS stimulation activates both the TLR4 and Notch signaling pathways in heart tissue. TLR4 and Notch pathway interaction enhanced the inflammatory response in the septic rat heart, leading to heart dysfunction and myocardial damage. However, only TLR4 inhibition with TAK242 but not Notch pathway inhibition with the γ-secretase inhibitor DAPT was able to prevent this [198]. Notch signaling is also studied in the context of balancing vascular homeostasis and in cardiovascular disorders [199,200]. Especially Notch-mediated regulation of the vascular tone gained attention [197,201,202,203]. In mouse aorta, sepsis leads to downregulation of Notch signaling and its effector genes and decreased contractile signaling performance. In part, the inducible nitric oxide synthase/nitric oxide (iNOS/NO) pathway is responsible for sepsis-induced down-regulation of Notch3. In contrast to the in vitro gained results in macrophages, systemic blocking of Notch signaling does not lead to a favorable aftereffect on sepsis-induced vascular hypo reactivity [204]. 

In a clinical observational study, plasma midkine, a small, cysteine-rich polypeptide and a multi-functional factor mainly secreted in embryogenesis but also participating in various key pathological processes [205] was reported to be elevated in sepsis. Levels were related to sepsis severity and the angiotensin-converting enzyme (ACE) system [206]. In a cecal ligation and puncture (CLP) sepsis mouse model, circulating and lung midkine was increased and associated with severe lung injury. Lung treatment with adeno-associated virus (AAV) held off midkine expression and mitigated acute lung injury. In an in vitro approach, Notch2 was found to engage in the midkine induced activation of ACE system and angiotensin II release. Moreover, Notch2 elicits vascular endothelial injury by angiotensin II-induced ROS production [207].

Opposingly, Notch signaling also exerts protective effects during acute systemic inflammation. A targeted screen of known major signaling pathways identified Notch as a negative regulator of stimulators of interferon genes (STING) signaling in macrophages [208]. In a mouse model, inhibition of Notch with a γ-secretase inhibitor during endotoxemia increased STING-dependent apoptosis of splenic CD4^+^ T cells. Furthermore, NICD blocked STING activation by preventing cyclic dinucleotides (CDN), the only known STING ligand, binding to STING. These findings underscore the central roles of Notch and STING signaling in CD4^+^ T cell apoptosis during acute systemic inflammation and reveal that Notch is a negative regulator of STING [209]. 

### 4.3. Targeting Notch Signaling as a Therapeutic Intervention for Sepsis and Beyond

Interactions of a few non-coding ribonucleic acids (RNA) with the Notch signaling pathways in a disease context are known [210]. Noncoding RNA (ncRNA) comprise of a diverse range of RNA species, including microRNAs (miRNA) and long noncoding RNA (lncRNA). MiRNA are of approximately 19–25 nucleotides in length and are involved in gene expression regulation. LncRNA are longer than 200 nucleotides and can activate or repress gene expression [211]. Several lncRNA and miRNA control the Notch signaling [210]. miRNA are also regulators of the immune response, with potential application in sepsis [212,213]. In plasma samples of sepsis patients, miR-150 was found to be downregulated and circulating miR-150 was identified as a prognostic marker in patients with critical illness and sepsis [214,215,216]. Likewise, miR-150 was reported to play a role in the pathogenesis of sepsis [217,218]. Deng et al. recently reported that miR-150 expression was reduced upon LPS administration. Furthermore, miR-150 relieved LPS-induced inflammatory response and apoptosis in RAW264.7 cells. This effect might be explained by the identification of Notch1 as a direct target of miR-150 [219]. 

Two other studies from Cao et al. and Mraz et al. described that miR-34a displayed a regulative capacity on Notch signaling pathway during immune response and inflammation [220,221]. Thus, Ge et al. found that the Notch-1 expression was decreased 24 h after LPS treatment while NF-κB was significantly increased. This is also reflected in the fact that pro-inflammatory cytokines were reduced, and anti-inflammatory cytokines were increased after intervening with miR-34a. In addition, Notch-1 mRNA and protein levels were increased under miR-34a, whereas NF-κB was downregulated, concluding that miR-34a regulating Notch-1/NF-κB signaling pathway can reduce endothelial damage caused by LPS [222].

MiR-146b is related to myocardial disease and has also been reported to have an effect in sepsis [223,224,225]. In a mouse model of septic cardiac dysfunction miR-146b was increased significantly in the myocardium. Upregulation of miR-146b suppresses IL-1β expression and apoptosis of the myocardium and Notch1 has been identified as a target gene of miR-146b. This verifies that in cardiomyocytes decreased miR-146b led to increased expression of Notch1, concluding that miR-146b protects cardiomyocytes against inflammation [226]. 

In different neoplastic conditions, several oncogenic or tumor suppressor lncRNAs have been recognized to interact with the Notch signaling pathway [227]. However, in the context of Sepsis, only one lncRNA has been described so far. In a sepsis mouse model, the lncRNA HOTAIRM1 (HOXA transcript antisense RNA myeloid-specific 1) is highly expressed in the late phase of the disease. Upregulation of HOTAIRM1 is crucial for the formation of an immunosuppressive environment and is induced via Notch/Hes1 activation. HOTAIRM1 promotes T cell exhaustion by increasing the number of regulatory T cells, programmed cell death protein 1 (PD-1) positive T cells as well as elevation of programmed death-ligand 1 (PD-L1). This leads to the conclusion that the Notch/Hes1/HOTAIRM1/HOXA1/PD-L1 axis is critical for sepsis-induced immunosuppression [228]. 

A variety of small inhibitory molecules targeting Notch have been investigated in inflammatory disease animal models [229,230,231]. Asiatic acid (AA, a triterpenoid) from *Centella asiatica* has been described to suppress TNF, IL-1β, and IL-6 expression via suppression of nucleotide-binding domain (NOD)-like receptor protein 3 (NLRP3) inflammasome activation [232]. Yuyun et al. investigated the potential contribution of Notch signaling to this process. In an endotoxemia mouse model, AA significantly improved survival. AA markedly reduced the extent of tissue damage (cellular necrosis, alveolar wall thickening and endothelial cell swelling in organs), neutrophil infiltration, and inhibited LPS-induced release of IL-1β and IL-6. RAW264.7 cells were used to investigate the underlying mechanisms and it has been found that after stimulation with LPS, AA notably inhibited Notch3 and Dll4. Furthermore, a close connection of Notch 3 signaling with the IL-6 promoter was revealed. AA mitigated the effect [233]. 

Another potential way to target the Notch signaling cascade is to interrupt the translocation of NICD by inhibiting γ-secretase with DAPT. γ-secretase inhibition down-regulates the expression of Notch1 and NF-κB. This reduces brain damage in middle cerebral artery occlusion in rats [234]. Normalized signaling via the Notch/HES-1 axis eliminates inflammation and thus protects the nervous system in a neurotoxicity mouse model [235]. Biliary atresia-induced mortality has been delayed and serum levels of proinflammation cytokines have been reduced as well [236]. In a rat CLP model, sepsis increased the expression of hippocampal NICD and poly (adenosine diphosphate [ADP]-ribose) polymerase-1 (PARP-1). Inhibiting γ-secretase with DAPT significantly decreases the level of NICD and PARP-1, reduces hippocampal neuronal apoptosis, weakens TNF release, and releases cognitive impairment. The neuroprotective effect of γ-secretase inhibition on neuronal death and memory impairment, could be a novel therapeutic approach to treat sepsis-associated encephalopathy or other sepsis-induced sequelae in the future [237]. γ-Secretase has now been proposed to be a therapeutic target in various cancers [238,239,240,241,242,243,244], immunologic disorders vasculitis [245], macular degeneration [246], diabetic nephropathy [115,247], ischemic reperfusion injury in the kidney [248], ischemic stroke [249], traumatic brain injury [250], hearing loss [251], and fibrosis [252]. However, with more than 90 known substrates of γ-secretase, the therapeutic approach must be further investigated. The biological role of γ-secretase in cleavage of substrates other than Notch has been ignored in several preclinical repurposing studies. For example, in a γ-secretase inhibitor (DAPT) Alzheimer’s disease trial, many participants noted hair color changes, apparently due to inhibition of tyrosinase, another γ-secretase substrate [253]. In further studies, additional Notch paralogs and VEGFR1 have been considered as targets [149,253,254]. Currently, no tools are readily available to perform simple yet detailed studies on the impact of γ-secretase cleavage on γ-secretase substrates other than amyloid precursor protein (APP) and Notch 1. Therefore, to deepen the understanding of the biological consequences of a γ-secretase inhibitor-based therapies, it will be important to develop such tools [255]. Given the fact that the biology of γ-secretase is complex, the current knowledge remains poor. People are quick to focus only on the easily observable areas of Notch and APP 1 and disregard the others mentioned above [256].

## 5. Conclusions

Regardless of the relative simplicity of the core cascade, the ability of the Notch signaling pathway to execute many functions depends on different control points that shape the pathway towards its action. The range and strength of Notch signaling is controlled by the tissue structures and the expression patterns of the Notch receptor and its ligands. Notch signaling interacts with other signaling cascades in the nucleus and conjointly induces a variety of effects. Although Notch ligands are transmembrane proteins, the range of the signal is limited, emphasizing cell architecture and tissue organization as certain factors. All regulation steps can be inflected to allow the pathways to adapt to the respective environment. Current research is dominated by structural investigation of key complexes associated with Notch signaling. Subsequently, the dynamics of the different complexes must be studied and how they can be modulated through environmental factors. These insights will help to predict transcriptional and physiological outcomes, but also to better understand the susceptibility to sepsis. The current understanding of the Notch signaling pathway in the context of sepsis and acute inflammatory responses provides a guide to where therapeutic interventions may be possible in the future. Including but not limited to, Notch modulation by non-coding RNA or suppression of γ-secretase via DAPT must be investigated further [219,222,228,237]. Modulation of Notch receptors at the molecular level provides future potential for therapies in acute inflammation and other Notch-related diseases. There are still open questions that need to be addressed. A future area of research will be to investigate how the signals are translated into specific and categorical transcriptional responses in the different cell types and environments. Possible therapeutic targets include ligands, receptors, regulators, and transcription factors depending on the respective tissue environment. 

## Data Availability

Not applicable.

## Abbreviations

AA	Asiatic acid
AAV	Adeno-associated virus
ACE	Angiotensin-converting enzyme
ADAM	A Dys integrin and metalloprotease
AKI	Acute Kidney Injury
Akt	Protein kinase B
Ang2	Angiopoietin 2
ASC	Antibody-secreting Cells
BAFF	B cell-activating Factor
BCL-1	B Cell Lymphoma 1 Protein
BCR	B cell Receptor
BM	Bone Marrow
BMP	Bone Morphogenetic Proteins
CBP	Cardiopulmonary Bypass
CD	Cluster of Differentiation
CDKN1A	Cyclin Dependent Kinase Inhibitor
CDN	Cyclic Dinucleotides
CLP	Cecal Ligation and Puncture
COX2	Cyclooxygenase 2
CSL	C-promoter-binding factor CBF-1
DAMPS	Damage-associated Molecular Patterns
DAPT	(N-[N-(3, 5-difluorophenacetyl)-l-alanyl]-s-phenylglycinet-butyl ester
DC	Dendritic Cells
DLL	Delta-like Ligand
DNA	Deoxyribonucleic Acid
EC	Endothelial Cell
EGF	Endothelial Growth Factor
GATA3	GATA Binding Protein 3
GFR	Glomerular Filtration Rate
GM-CSF	Granulocyte-Macrophage Colony-stimulating Factor
HOTAIRM1	HOXA transcript antisense RNA myeloid-specific 1
HSC	Hematopoietic Stem Cells
HSPC	Hematopoietic Stem Progenitor Cells
ICU	Intensive Care Unit
IFN	Interferon
IL	Interleukin
iNOS/NO	Inducible Nitric oxide Synthase/Nitric oxide
JNK	C-Jun-N-terminal Kinase
lcncRNA	Long noncoding RNA
LDL	Low density lipoprotein
Lfng	Lunatic Fringe
LPS	Lipopolysaccharide
Maml1	Mastermind Like Transcriptional Coactivator 1
MAPK	Mitogen-activated Protein Kinases
M-CSF	Macrophage Colony-stimulating Factor
Mfng	Manic Fringe
miRNA	micro-RNA
MZB	Marginal Zone B cells
ncRNA	noncoding RNA
NCSC	Neural crest stem cells
NF-κB	Nuclear Factor-κB
NICD	Notch Intracellular Domain
NLRP3	Nucleotide-binding domain (NOD)-like receptor protein 3
PAMPS	Pathogen-associated Molecular Patterns
PARP	Poly (adenosine diphosphate [ADP]-ribose) Polymerase-1
PD-1	Programmed Cell Death Protein 1
pDC	plasmacytoid DC
PD-L1	Programmed Death-Ligand 1
POFUT	Protein O-fucosyl transferase 1
PTCRA	Pre-T cell Antigen Receptor alpha
RBP-J	Recombination Signal-Binding Protein Jkappa
RNA	Ribonucleic Acid
ROS	Reactive Oxygen Species
SIRS	Systemic Inflammatory Response Syndrome
SLE	Systemic lupus erythematosus
SOCS3	Suppressor of Cytokine Signaling 3
SMAD	Suppressor of Mothers against Decapentaplegic
SSc	Systemic sclerosis
STING	Stimulators of Interferon genes
T-ALL	T-lineage Acute Lymphoblastic Leukemia
TAM	Tumor-associated Macrophages
TCR	T cell Receptor
Th1	Type 1 T helper cell
Th17	T helper 17 cell
Th2	Type 2 T helper cell
TLR	Toll-like-Receptor
TNF	Tumor Necrosis Factor
VEGF	Vascular Endothelial Growth Factor
VEGFR	Vascular Endothelial Growth Factor Receptor
